# Laser-Assisted Orthodontic Tooth Movement in Saudi Population: A Prospective Clinical Intervention of Low-Level Laser Therapy in the 1st Week of Pain Perception in Four Treatment Modalities

**DOI:** 10.1155/2019/6271835

**Published:** 2019-10-20

**Authors:** Mohammad Khursheed Alam

**Affiliations:** Orthodontic Division, Preventive Dentistry Department, College of Dentistry, Jouf University, Sakaka, Al Jouf, Saudi Arabia

## Abstract

**Background:**

This first-in-human study in Saudi orthodontic patients has evaluated the role of low-level laser therapy (LLLT) in pain perception (PP). The outcome of single application of LLLT with 4 different treatment modalities (TM) on PP are evaluated following orthodontic bracket bonding on maxilla.

**Materials and Methods:**

A prospective clinical intervention with implementation of parallel technique in each group, 32 orthodontic patients with ectopic canine requiring fixed orthodontic appliance were enrolled and randomly allocated to the 4 groups: LLLT + self-ligating (SL) bracket, LLLT + conventional (Conv.) bracket, non-LLLT + SL bracket, and non-LLLT + Conv. bracket. Orthodontic bracket bonding from 1st molar to 1st molar and superelastic 0.012 inch NiTi were applied for the maxilla. For each patient, maxillary 1st molar to molar received a single application of LLLT using a 940 nm Ga-Al-As laser device on 5 different points labially/buccally and palatally. Main outcome measure was the degree of PP score during the 1st week of orthodontic tooth movement (OTM) after 4 hours, 24 hours, 3 days, and 7 days of both LLLT and non-LLLT treatment applications. A questionnaire with an 11-point numeric rating scale (NRS) was used for PP.

**Results:**

Mean ± SD of PP in the LLLT + SL group was 3.33 ± 1.4, 3.58 ± 1.06, 2.31 ± 0.67, and 1.89 ± 0.54 in 4 hours, 24 hours, 3 days, and 7 days, respectively. Compared to all 4 TM groups, LLLT groups showed better PP. More statistically significant differences were found in LLLT groups. No harms were encountered.

**Limitations:**

The intervention provider and the patient were not blinded to the intervention.

**Conclusion:**

The LLLT + SL group revealed significantly promising benefits on PP during OTM.

## 1. Introduction

LLLT has promising benefits on OTM. It has been revealed that 940 nm diode LLLT significantly increase osteoblast cells during their proliferation and differentiation stages. Thus, it induces bone remodelling by stimulating osteogenesis [1, 2]. Quality bone regeneration leads to better bone remodelling which is essential for OTM. Findings suggest that LLLT can enhance the velocity of tooth movement and improve the quality of bone remodelling during OTM [3].

In a systematic review of animal studies on acceleration of OTM, using noninvasive techniques suggested for further research studies to establish protocols to use them clinically with conviction [4]. Based on the literature review, it was concluded as the use of noninvasive techniques are beneficial and promising [5].

Research in relation to pain and its control in clinical situations did not progress/advance much as one of the major impeding factors for such a delay was our incapability in measuring pain effectively. A spectrum of studies were carried out considering pain as signals of tissue injury at one end and as a completely subjective phenomenon.

The effect of a single dose of LLLT on spontaneous and chewing pain after the placement of elastomeric separators on OTM [6], PP with the velocity of OTM and the PP with it using self-ligating (SL) brackets, [7] and the analgesic effect of a single application of LLLT on spontaneous pain and pain on chewing after placement of initial archwires [8] have been investigated and revealed promising results. Hence, it can be concluded that a single dose of LLLT considerably lowered postoperative pain on OTM [6–8].

Studies have revealed the usefulness of LLLT in the reduction of PP immediately following the placement of separators [6] and initial archwire [7, 8]. However, there were no studies indicating the exact role of LLLT in alleviating PP and OTM. Thus, this study will seek to uncover the level of PP with the velocity of OTM and the PP in LLLT and non-LLLT groups with SL brackets or Conv. brackets.

## 2. Materials and Methods

After obtaining the approval from the Ethical Committee of Jouf University (LCBE#4-22-2/40), which complies with the Declaration of Helsinki, a written informed consent was obtained from all the subjects (one of the parents, either father and/or mother or legal guardian for adolescent subjects). This study was designed and conducted according to the guidelines of Strengthening the Reporting of Observational Studies in Epidemiology (STROBE).

**Figure 1:**
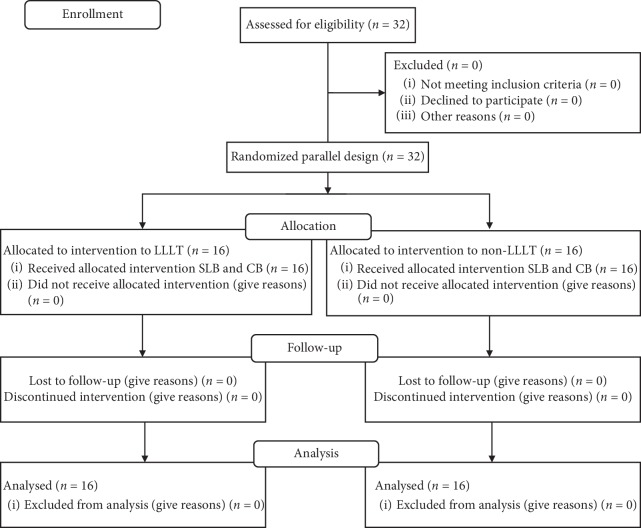
Consort flow diagram.

Thirty-two healthy orthodontic patients of Saudi ethnic background with ages between 14 and 25 years were selected for the study (Figure 1). Based on TM, all patients are randomly divided into 4 groups: LLLT + SL, non-LLLT + SL, LLLT + Conv., and non-LLLT + Conv. groups. Inclusion criteria, exclusion criteria, PICOS, study groups, 4 different TM, number of subjects in each group, armamentarium used, orthodontic treatment, laser application, PP assessment, and statistical analysis are detailed in Table 1.

## 3. Results

**Figure 2:**
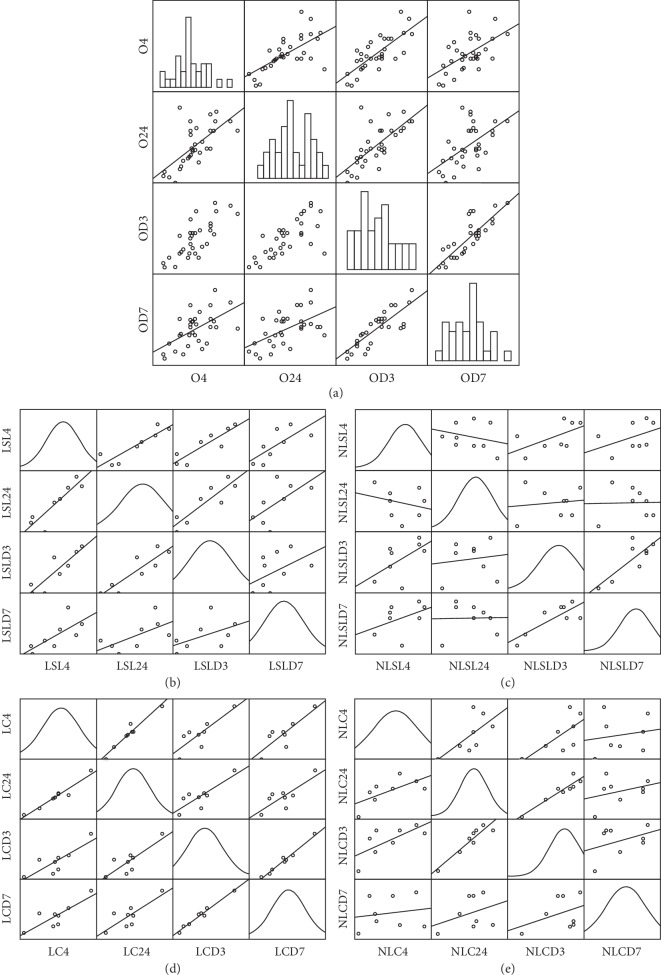
Scatterplot matrix.

Figure 2 shows collection of scatterplots called scatterplot matrix. Each scatterplot shows the relationship between a pair of PP outcome in relation to OTM treatment time. Figure 2(a) is an example that uses the total data of 4 different PP outcomes in relation to OTM treatment time, which contains data for PP measurements for 32 subjects. This study explores the following questions: is there a relationship between any pair of PP outcome in relation to OTM treatment time and which pair has the strongest relationship. There are 8 possible pairs. All 8 pairs of variables are positively correlated. The strongest relationship appears to be between 4 hours and 24 hours. Figures 2(b)–2(e) show collection of scatterplots of LLLT + SL, non-LLLT + SL, LLLT + Conv., and non-LLLT + Conv. groups, respectively. LLLT + SL and LLLT + Conv. groups show maximum pairs of variables are positively correlated compared to non-LLLT groups.

Tables 2 and 3 show the results of Kruskal–Wallis *H* test, all 4 TM groups in relation to PP outcome with OTM treatment time.

  PP after 4 hours was LLLT + SL < LLLT + Conv.<non-LLLT + SL < non-LLLT + Conv.
  PP after 24 hours was LLLT + SL < LLLT + Conv.<non-LLLT + Conv. < non-LLLT + SL.
  PP after 3 days was LLLT + SL < non-LLLT + SL < LLLT + Conv. < non-LLLT + Conv.
  PP after 7 days was LLLT + SL < non-LLLT + SL < LLLT + Conv. < non-LLLT + Conv.

Table 4 shows the results of step-by-step pairwise group comparison by Mann–Whitney *U* test to explore the exact differences between groups. Table 4(a) shows the results of LLLT + SL vs. non-LLLT + SL. LLLT + SL shows significantly better results in 24 hours (*p*=0.011), 3 days (*p*=0.013), and 7 days (*p*=0.001). Table 4(b) shows the results of LLLT + SL vs. LLLT + Conv. LLLT + SL shows significantly better results in 3 days (*p*=0.021) and 7 days (*p*=0.002). Table 4(c) shows the results of LLLT + SL vs. non-LLLT + Conv. LLLT + SL shows significantly better results in 4 hours (*p*=0.040), 24 hours (*p*=0.006), 3 days (*p*=0.002), and 7 days (*p*=0.001). Table 4(d) shows the results of non-LLLT + SL vs. LLLT + Conv. LLLT + Conv. shows significantly better results in 24 hours (*p*=0.024). Table 4(e) shows the results of non-LLLT + SL vs. non-LLLT + Conv. Non-LLLT + SL shows significantly better results in 3 days (*p*=0.013). Table 4(f) shows the results of LLLT + Conv. vs. non-LLLT + Conv. LLLT + Conv. shows significantly better results in 24 hours (*p*=0.031).

**Figure 3:**
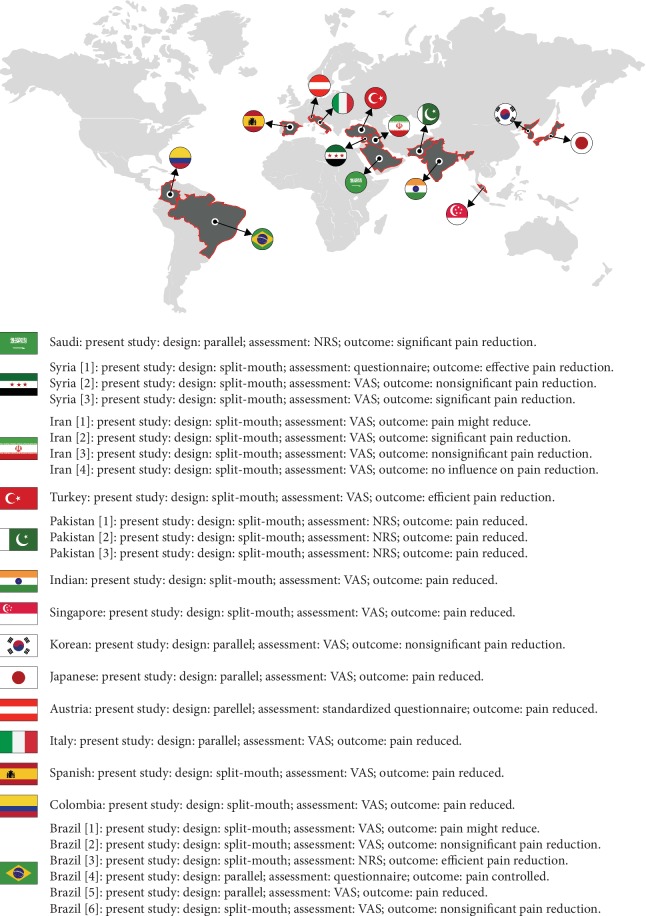
Present study and global research outcome of laser + OTM + pain perception.

Figure 3 shows global mapping of 26 studies [6–30] from 14 different countries.

## 4. Discussion

Based on extensive literature search, this is first-in-human study that evaluated 4 different TM outcomes on Saudi ethnic orthodontic patient. In this study, prospective evaluation of the consequences of a single application of LLLT on pain consecutively after 4 hours, 24 hours, 3 days, and 7 days after orthodontic treatment was conducted in parallel study design. Parallel study design is efficient and has uniformity in allocation segment and has no carry-across effects or contamination which is the main disadvantage for split-mouth studies [19–22, 28, 29].

An infrared radiation with a wavelength of 940 nm [6–8] and a LLLT having a wavelength close to the lower end of the infrared electromagnetic spectrum were used in this study. It is believed that the deeper penetration of infrared radiation is because it has a low absorption coefficient in haemoglobin and water. It is a well-established fact that the penetration of irradiated tissues is greater by the infrared radiation than lasers in visible spectrum and could probably reach cortices and alveolar bone [28]. Laser beam was applied over 5 specific points facially and palatally as suggested by Qamruddin et al. [6–8] and Tortamano et al. [27].

Present study and 25 different global studies' results of 14 different countries are highlighted in Figure 3. Global mapping of 26 studies [6–30] from 14 different countries that evaluated the effects of laser on OTM and PP showed divergent results. Results from 14 countries revealed neutral [12, 15], nonsignificant reduction [10, 14, 19, 26, 30], pain reduction [9, 16–18, 20–25, 27–29], and significantly lessened pain [6–8, 11, 13] on OTM (Figure 3). Different studies used different ways to measure pain perception; among them, VAS [10–20, 22–26, 29, 30], NRS [6–8, 27], and self-designed questionnaire [9, 21, 28] were common. This study used NRS for PP. It was possible to gather information telephonically since NRS has added advantages of being verbally administered as a very potent assessment tool and was shown to correspond and correlate with visual analogue scale [6–8, 31]. The present study used parallel design and NRS and revealed significantly better results in relation to LLLT TM. LLLT + SL TM was observed as the best TM among all four.

Various studies have investigated the role of LLLT on a variety of biological processes [1–5], and it was established that it reduces pain effectively [6–9, 11, 13, 16–18, 20–25, 27–29, 32]. Although the exact mechanism is still obscure, it has been suggested that it might alleviate pain by stimulating the nerve cell, stabilizing the membrane potentials and the release of neurotransmitters [21]. Lasers have been used effectively to decrease the postadjustment pain in orthodontics [18, 20, 21]. Although the application of LLLT in clinical dentistry has been widely practised [27], its exact mechanism of action is indeterminate. However, it has been proposed that it may not only stimulate the nerve cells and lymphocytes with the resultant release of neurotransmitter substances but also may stop nerve signalling and thus decrease pain perception [27]. It has also been suggested that it increases blood circulation which clears off the pain-producing mediators of inflammation and increases the reparative process through biostimulation [20]. LLLT was effectively used in periodontal tissues for pain relief [18, 19, 21, 27]. It has been suggested that it reduces pain by preventing the release of arachidonic acid thus reducing the levels of prostaglandin E2 [30] and secondly by the release of beta-endorphin [33]. During the procedure, none of the patients complained of any discomfort or heat suggesting a very low energy output by the laser and that LLLT is a noninvasive, nonthermal procedure [6–8].

The present study reports in favour of SL bracket. These results coincide with results reported by Qamruddin et al. [7]. The advantages of utilizing passive SL brackets with respect to loss of anchorage, associated pain, and the rate of tooth movement are contentious [7]. Hence, it better to thoroughly re-evaluate the advantages of SL brackets and subsequently the added advantages of LLLT along with SL brackets. According to Flaming and Johal [33], there is less pain and decreased levels of substance P in GCF noted after 24 hours of insertion of archwires with SL brackets as compared to conventional brackets. The level of substance P is a good indicator of pain; hence, their study strengthened the opinion of lower pain with lighter forces.

Gender disparities were not considered in this report due to inequality in subject distribution between male and female. Female patients were more than the male. Qamruddin et al. [7] found nonsignificant gender-based dissimilarity in PP at any phase of the study. Within the limitation, further studies are required with long-term follow-up and are indispensable to strengthen the findings of the present study with larger sample size and assess the precise benefits of LLLT on bone remodeling and overall quality of life.

## 5. Conclusion

This clinical prospective intervention revealed LLLT has promising benefit among all 4 treatment modalities, and LLLT + SL results the best and LLLT + Conv. as the 2nd best in lessened pain perception during the 1st week of OTM. Lastly, comparing the efficacy among 2 bracket systems, SL lessened pain perception better compared to Conv.

## Figures and Tables

**Table 1:** Subjects and methodology in detail.

Selection guideline	Inclusive	Exclusive		
	Angle class I or II or III malocclusion with ectopic maxillary canine requiring space creation or extraction of first premolar.	Patient on long-term medication, craniofacial anomalies/malformation, with parafunctional habits, temporomandibular joint dysfunction, multiple missing teeth, and periodontally compromised		

Population	Patients in orthodontic treatment			
Intervention	Laser-assisted orthodontic tooth movement			
Comparison	Nonlaser-assisted orthodontic tooth movement			
Outcome	Laser-assisted orthodontic tooth movement			
Study design	Prospective clinical intervention			
Sample size calculation	G*∗*Power software version 3.0.10 with power 80%, *α* 0.05, and effect size (*d*) 0.22 was used. Hence, the total sample size intended for this research was 32, each group required minimum of 8 subjects.			

Study groups	Laser		Nonlaser	
Treatment modalities	Self-ligating	Conventional	Self-ligating	Conventional
Number of subjects	8	8	8	8
Age of subjects	Between 14 and 25.			

Armamentarium	The laser unit was a 940 nm aluminum-gallium-arsenide (Al-Ga-As) diode laser (iLase; Biolase, Irvine, CA, USA) set on continuous mode with power at 100 mW. The diameter of the optical fiber tip was 0.04 cm^2^, the energy density was calculated to be 7.5 J/cm2 for each point, and total energy density was 75 J per tooth.			
Orthodontic treatment	For all patients, treatment has been commenced by bonding the upper arch with preadjusted edgewise 0.022 inch slot MBT prescription brackets, Agility® self-ligating bracket system (Franklin, USA), and ortho organizers conventional type bracket system (Carlsbad, CA, USA). Alignment and leveling started using 0.012 inch superelastic nickel-titanium (NiTi) wire followed by 0.014, 0.016, and 0.018 in NiTi wires, changed at 4-week interval between each wire.			
Laser application	Laser applied on gingival mucosa for 3 seconds each on 5 points labially/buccally and palatally per tooth, starting from central incisor to the first molar. These points were mesial and distal over the cervical-third of the root and the middle of the root and also mesial and distal over the apical-third of the root. The fiber tip of the laser was in close but light contact with the surface of the gingival tissues and held perpendicular to the mucosa overlying the roots of teeth.			
Pain perception	Numerical rating scale (NRS) questionnaire was used to measure pain intensity. After orthodontic bracket bonding and/or application of laser, these questionnaires were given to the participants to be completed at home and returned at the following appointment. The participants were asked to record pain after 4 hours, 24 hours, 3 days, and 7 days. In addition, telephone calls/messages were made at day-3 and day-7 to ensure accurate collection of data.			
Statistical analysis	IBM SPSS Statistics version 22.0 (IBM Co., Armonk, NY, USA) was used to analyze the data. Descriptive analysis was performed to obtain the mean values of pain in among 4 groups. Since the distribution of data was not normal, series of Mann–Whitney *U* test was used to compare the level of pain between 2 groups. A scatterplot matrix with all scatterplots is presented. Kruskal–Wallis *H* test was performed to see the differences among 4 groups.			

**Table 2:** Kruskal–Wallis *H* Test comparison among all groups.

Groups	Mean	SD	Minimum	Maximum	Mean rank
After 4 hours					
LLLT + SL	3.325	1.391	1.500	5.200	11.190
NLLLT + SL	4.463	1.041	2.900	5.800	18.310
LLLT + Conv.	3.863	1.144	2.000	5.900	14.380
NLLLT + Conv.	5.225	1.694	3.000	7.800	22.130
Total	4.219	1.463	1.500	7.800	**p=0** **.104**
After 24 hours					
LLLT + SL	3.575	1.059	2.000	5.000	9.560
NLLLT + SL	5.638	1.377	3.500	7.500	22.250
LLLT + Conv.	4.088	1.198	2.400	6.500	11.940
NLLLT + Conv.	5.600	1.196	3.200	7.200	22.250
Total	4.725	1.479	2.000	7.500	**p=0** **.006**
After 3 days					
LLLT + SL	2.313	0.669	1.500	3.200	7.190
NLLLT + SL	3.538	0.825	2.200	4.500	16.690
LLLT + Conv.	3.675	1.250	2.200	6.200	16.880
NLLLT + Conv.	4.863	1.107	2.600	6.000	25.250
Total	3.597	1.314	1.500	6.200	**p=0** **.002**
After 7 days					
LLLT + SL	1.188	0.541	0.500	2.200	4.940
NLLLT + SL	2.800	0.545	1.800	3.400	17.940
LLLT + Conv.	3.188	1.197	1.600	5.500	20.560
NLLLT + Conv.	3.450	0.898	2.300	4.600	22.560
Total	2.656	1.198	0.500	5.500	**p=0** **.001**

**Table 3:** Continuous step-by-step comparison between 2 groups using Mann–Whitney *U* test.

Groups	Mean rank	Sum of ranks	Mann–Whitney *U*	Wilcoxon *W*	*p* value
LLLT + SL vs. NLLLT + SL					
After 4 hours					
LLLT + SL	6.50	52.00	16.00	52.00	0.092
NLLLT + SL	10.50	84.00			
After 24 hours					
LLLT + SL	5.50	44.00	8.00	44.00	0.011
NLLLT + SL	11.50	92.00			
After 3 days					
LLLT + SL	5.56	44.50	8.50	44.50	0.013
NLLLT + SL	11.44	91.50			
After 7 days					
LLLT + SL	4.69	37.50	1.50	37.50	0.001
NLLLT + SL	12.31	98.50			

LLLT + SL vs. LLLT + Conv.					
After 4 hours					
LLLT + SL	7.63	61	25.00	61.00	0.462
LLLT + Conv.	9.38	75			
After 24 hours					
LLLT + SL	7.81	62.5	26.50	62.50	0.563
LLLT + Conv.	9.19	73.5			
After 3 days					
LLLT + SL	5.75	46	10.00	46.00	0.021
LLLT + Conv.	11.25	90			
After 7 days					
LLLT + SL	4.75	38	2.00	38.00	0.002
LLLT + Conv.	12.25	98			

LLLT + SL vs. NLLLT + Conv.					
After 4 hours					
LLLT + SL	6.06	48.5	12.50	48.50	0.040
NLLLT + Conv.	10.94	87.5			
After 24 hours					
LLLT + SL	5.25	42	6.00	42.00	0.006
NLLLT + Conv.	11.75	94			
After 3 days					
LLLT + SL	4.88	39	3.00	39.00	0.002
NLLLT + Conv.	12.13	97			
After 7 days					
LLLT + SL	4.5	36	0.00	36.00	0.001
NLLLT + Conv.	12.5	100			

NLLLT + SL vs. LLLT + Conv.					
After 4 hours					
NLLLT + SL	9.44	75.5	24.50	60.50	0.429
LLLT + Conv.	7.56	60.5			
After 24 hours					
NLLLT + SL	11.19	89.5	10.50	46.50	0.024
LLLT + Conv.	5.81	46.5			
After 3 days					
NLLLT + SL	8.69	69.5	30.50	66.50	0.874
LLLT + Conv.	8.31	66.5			
After 7 days					
NLLLT + SL	7.5	60	24.00	60.00	0.398
LLLT + Conv.	9.5	76			

NLLLT + SL vs. NLLLT + Conv.					
After 4 hours					
NLLLT + SL	7.38	59	23.00	59.00	0.342
NLLLT + Conv.	9.63	77			
After 24 hours					
NLLLT + SL	8.56	68.5	31.50	67.50	0.958
NLLLT + Conv.	8.44	67.5			
After 3 days					
NLLLT + SL	5.56	44.5	8.50	44.50	0.013
NLLLT + Conv.	11.44	91.5			
After 7 day					
NLLLT + SL	7.13	57	21.00	57.00	0.245
NLLLT + Conv.	9.88	79			

LLLT + Conv. vs. NLLLT + Conv.					
After 4 hours					
LLLT + Conv.	6.44	51.5	15.50	51.50	0.083
NLLLT + Conv.	10.56	84.5			
After 24 hours					
LLLT + Conv.	5.94	47.5	11.50	47.50	0.031
NLLLT + Conv.	11.06	88.5			
After 3 days					
LLLT + Conv.	6.31	50.5	14.50	50.50	0.066
NLLLT + Conv.	10.69	85.5			
After 7 days					
LLLT + Conv.	7.81	62.5	26.50	62.50	0.563
NLLLT + Conv.	9.19	73.5			

**Table 4:** Step-by-step comparison between 2 groups using Mann–Whitney *U* test

Groups	Mean rank	Sum of ranks	Mann–Whitney *U*	Wilcoxon *W*	*p* value
(a) LLLT + SL vs. NLLLT + SL					
After 4 hours					
LLLT + SL	6.50	52.00	16.00	52.00	0.092
NLLLT + SL	10.50	84.00			
After 24 hours					
LLLT + SL	5.50	44.00	8.00	44.00	0.011
NLLLT + SL	11.50	92.00			
After 3 days					
LLLT + SL	5.56	44.50	8.50	44.50	0.013
NLLLT + SL	11.44	91.50			
After 7 days					
LLLT + SL	4.69	37.50	1.50	37.50	0.001
NLLLT + SL	12.31	98.50			

(b) LLLT + SL vs. LLLT + Conv.					
After 4 hours					
LLLT + SL	7.63	61	25.00	61.00	0.462
LLLT + Conv.	9.38	75			
After 24 hours					
LLLT + SL	7.81	62.5	26.50	62.50	0.563
LLLT + Conv.	9.19	73.5			
After 3 days					
LLLT + SL	5.75	46	10.00	46.00	0.021
LLLT + Conv.	11.25	90			
After 7 days					
LLLT + SL	4.75	38	2.00	38.00	0.002
LLLT + Conv.	12.25	98			

(c) LLLT + SL vs. NLLLT + Conv.					
After 4 hours					
LLLT + SL	6.06	48.5	12.50	48.50	0.040
NLLLT + Conv.	10.94	87.5			
After 24 hours					
LLLT + SL	5.25	42	6.00	42.00	0.006
NLLLT + Conv.	11.75	94			
After 3 days					
LLLT + SL	4.88	39	3.00	39.00	0.002
NLLLT + Conv.	12.13	97			
After 7 days					
LLLT + SL	4.5	36	0.00	36.00	0.001
NLLLT + Conv.	12.5	100			

(d) NLLLT + SL vs. LLLT + Conv.					
After 4 hours					
NLLLT + SL	9.44	75.5	24.50	60.50	0.429
LLLT + Conv.	7.56	60.5			
After 24 hours					
NLLLT + SL	11.19	89.5	10.50	46.50	0.024
LLLT + Conv.	5.81	46.5			
After 3 days					
NLLLT + SL	8.69	69.5	30.50	66.50	0.874
LLLT + Conv.	8.31	66.5			
After 7 days					
NLLLT + SL	7.5	60	24.00	60.00	0.398
LLLT + Conv.	9.5	76			

(e) NLLLT + SL vs. NLLLT + Conv.					
After 4 hours					
NLLLT + SL	7.38	59	23.00	59.00	0.342
NLLLT + Conv.	9.63	77			
After 24 hours					
NLLLT + SL	8.56	68.5	31.50	67.50	0.958
NLLLT + Conv.	8.44	67.5			
After 3 days					
NLLLT + SL	5.56	44.5	8.50	44.50	0.013
NLLLT + Conv.	11.44	91.5			
After 7 days					
NLLLT + SL	7.13	57	21.00	57.00	0.245
NLLLT + Conv.	9.88	79			

(f) LLLT + Conv. vs. NLLLT + Conv.					
After 4 hours					
LLLT + Conv.	6.44	51.5	15.50	51.50	0.083
NLLLT + Conv.	10.56	84.5			
After 24 hours					
LLLT + Conv.	5.94	47.5	11.50	47.50	0.031
NLLLT + Conv.	11.06	88.5			
After 3 days					
LLLT + Conv.	6.31	50.5	14.50	50.50	0.066
NLLLT + Conv.	10.69	85.5			
After 7 days					
LLLT + Conv.	7.81	62.5	26.50	62.50	0.563
NLLLT + Conv.	9.19	73.5			

## Data Availability

The data used to support the findings of this study are included in the article.
